# Arbuscular mycorrhizal fungi suppress ammonia-oxidizing bacteria but not archaea across agricultural soils

**DOI:** 10.1016/j.heliyon.2024.e26485

**Published:** 2024-02-20

**Authors:** Daquan Sun, Martin Rozmoš, Michala Kotianová, Hana Hršelová, Jan Jansa

**Affiliations:** Laboratory of Fungal Biology, Institute of Microbiology, Czech Academy of Sciences, Vídeňská 1083, 14220 Praha 4, Czech Republic

**Keywords:** Arbuscular mycorrhizal (AM) fungi, Ammonia-oxidizing microorganisms (AO), Quantitative real-time PCR (qPCR), Agricultural soils, Land use, Cropland and grassland

## Abstract

Arbuscular mycorrhizal (AM) fungi are supposedly competing with ammonia-oxidizing microorganisms (AO) for soil nitrogen in form of ammonium. Despite a few studies directly addressing AM fungal and AO interactions, mostly in artificial cultivation substrates, it is not yet clear whether AM fungi can effectively suppress AO in field soils containing complex indigenous microbiomes. To fill this knowledge gap, we conducted compartmentalized pot experiments using four pairs of cropland and grassland soils with varying physicochemical properties. To exclude the interference of roots, a fine nylon mesh was used to separate the rhizosphere and mesh bags, with the latter being filled with unsterile field soils. Inoculation of plants with AM fungus *Rhizophagus irregularis* LPA9 suppressed AO bacteria (AOB) but not archaea (AOA) in the soils, indicating how soil nitrification could be suppressed by AM fungal presence/activity. In addition, in rhizosphere filled with artificial substrate, AM inoculation did suppress both AOB and AOA, implying more complex interactions between roots, AO, and AM fungi. Besides, we also observed that indigenous AM fungi contained in the field soils eventually did colonize the roots of plants behind the root barrier, and that the extent of such colonization was higher if the soil has previously been taken from cropland than from grassland. Despite this, the effect of experimental AM fungal inoculation on suppression of indigenous AOB in the unsterile field soils did not vanish. It seems that studying processes at a finer temporal scale, using larger buffer zones between rhizosphere and mesh bags, and/or detailed characterization of indigenous AM fungal and AO communities would be needed to uncover further details of the biotic interactions between the AM fungi and indigenous soil AO.

## Graphical abstract

**Figure:**
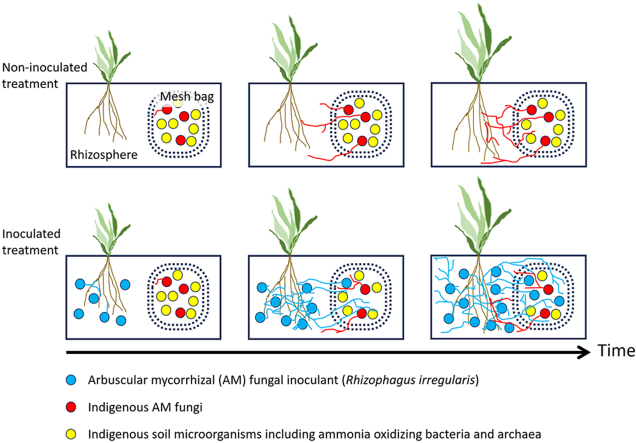

## 1 Introduction

Nitrogen (N) fertilizers have revolutionized agriculture by enhancing crop yields [1,2], but their excessive use has also resulted in serious environmental issues, such as elevated greenhouse gas (chiefly the N_2_O) emissions and water eutrophication (mainly by nitrates, which are used as fertilizers as such or which are formed from ammonium ions by nitrification) [3,4]. Arbuscular mycorrhizal (AM) fungi, which form symbiotic associations with most land plant species and are implicated in plant acquisition of mineral nutrients such as phosphorus and/or N, offer a promising strategy for improving plant mineral nutrition [[5], [6], [7]], fertilizers use efficiency and reduction of nutrient losses [8]. Moreover, recent investigations have revealed that AM fungi also possess the ability to mitigate N_2_O emissions through a variety of mechanisms, such as the transportation of NH_4_^+^-N to host plants via intricate hyphal networks [9], the modulation of denitrification activity in soil [10], and the regulation of NH_3_ and N_2_O emissions by enhancing plant N uptake, reducing N availability in soil [11], and altering soil bacterial communities [12,13]. These findings highlight the potential of AM fungi to promote sustainable agriculture by reducing the environmental impact of N fertilizers.

Nitrification, a crucial process in the soil N cycling, involves the biological oxidation of ammonia to nitrite followed by the oxidation of nitrite to nitrate [14]. This process is usually rate-limiting by oxidation of ammonia through ammonia-oxidizing microorganisms (AO), which include both ammonia-oxidizing bacteria (AOB) and ammonia-oxidizing archaea (AOA) [[15], [16], [17], [18]]. Yet, the conversion of ammonia to nitrite and nitrate increases N mobility and leachability and fuels denitrification and gaseous N losses [19]. The abundance of AO is therefore a critical determinant of ammonia oxidation rates and has a significant impact on ecosystem productivity, potential N leaching, and nitrous oxide emissions [20,21]. The AM fungi usually take up ammonium from soil to cover their own metabolic needs and for trading it against plant C in forms of hexoses and/or fatty acid with the host plant [[22], [23], [24]]. Ammonia-oxidizing microorganisms require ammonia as a fuel for driving nitrification process, which gains them energy [17,18]. This implies putative competition for this substrate between AM fungi and AO [25]. However, recent findings have challenged this assumption. Reference [26] showed that indigenous AM fungi increased the abundance of AO bacteria while reducing nitrous oxide emissions after urea application. Further, AM fungi were shown to alter inorganic N pools but without affecting AOB or AOA abundances in N-rich soils [27]. Recently, it has also been tested, by comparing addition of living AM inoculum vs. AM fungal necromass, whether any AM fungal metabolites could act as biological nitrification inhibitors, yet without conclusive support for such a scenario [28]. These results collectively convey the message that the relationship between AM fungi and AO is probably complex and context-dependent, and that other factors beyond competition for ammonia may play a role in shaping this biotic interaction.

The effect of AM fungi on AO in agricultural soils remains poorly understood. Despite of existing evidence for AO abundance (particularly with respect to the AOB) inhibited by AM fungal inoculation, previous studies mainly used artificial potting substrates and mock soil microbial communities [29,30] instead of employing field soils containing their complex indigenous microbiomes. Besides, agricultural soil use can lead to differences in soil N content and AM fungal community composition [31], which may in turn affect indigenous AO abundance and/or community composition. For instance, cropland soils tend to have higher N inputs and mineral N availabilities as compared to grasslands, implying possibly higher rates of N cycling [32]. Furthermore, environmental filtering of indigenous AM fungal communities due to land use has been described previously [31,33,34], suggesting that the interaction between AM fungi and AO may depend on soil legacy.

**Fig. 1:**
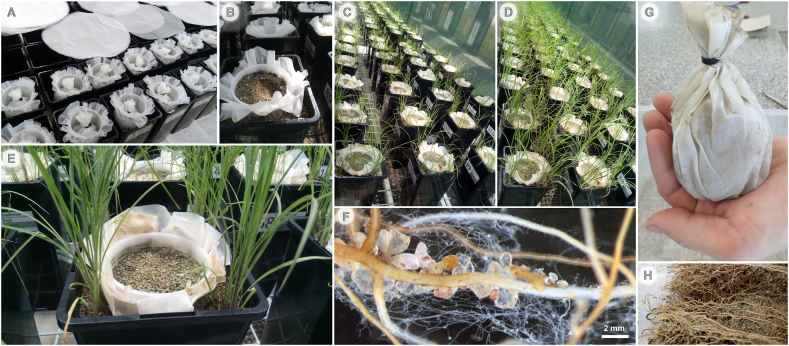
A compartmented cultivation system restricting access of roots to the cylinder placed in the center of the pots by a 40-μm mesh (nylon fabric) enwrapping the plastic cylinder. The cylinders were filled with mesh bags made of the same nylon fabric and containing sterile or unsterile field soils in Experiment 1 or Experiment 2, respectively (A–B). *Andropogon gerardii* roots are confined to the corners of each pot (C–E), creating a rhizosphere compartment outside the cylinders. Arbuscular mycorrhizal (AM) fungal hyphae growing out of the roots (F) can get access to the mesh bags (G). There was a dense root system developing outsides of the cylinders (H), whereas no roots were observed inside the cylinders.

To investigate the impact of AM fungi on AO (specifically, on the abundance of AOB and AOA), we conducted compartmentalized pot experiment, similar to a previous study [35], using four pairs of cropland and grassland soils with varying physicochemical properties. The soils were filled in mesh bags provided into a root-free zone of the pots, beyond a root barrier (Fig. 1). Our aim was to determine whether AM fungi could suppress AO oxidizers in both rhizosphere (considering root influence) and in the mesh bags (devoid of host plant roots). To achieve this objective, we formulated two specific experimental hypotheses: H1: AM fungi suppress indigenous AO across different agricultural soils; and H2: Effect of AM fungi on AO microorganisms may depend on soil legacy (cropland and grassland).

## 2 Materials and methods

### 2.1 Agricultural soil collection

Agricultural soils were collected on June 7th, 2022, from four locations including Drahohuz (50.53′N, 14.33′E), Encovany (50.53′N, 14.26′E), Malíč (50.54′N, 14.09′E), and Svařenice (50.50′N, 14.32′E) in Czechia. We collected pairs of agricultural soils (cropland and grassland) at each location to represent different soil uses. The details of the sampling sites (including their exact coordinates) are included in the Supplemental Material (see the Supplemental Material – Experimental timeline sheet). To ensure sample homogeneity and to remove stones and large roots/organic debris from naturally wet soils, the samples were sieved to pass through an 8 mm stainless steel sieve. Two kilograms of each sieved soil was γ-irradiated (using ^60^Co source, 25 kGy) at Bioster (Veverská Bítýška, Czechia), whereas another two kg of each soil were stored without any treatment, just cooled at 4 °C until the pot experiments (see below) started, which was approximately 1 week from field sampling. The basic physico-chemical properties of the field soils (sterilized or not) are presented in the Supplemental Material (Substrate and soil properties sheet).

### 2.2 Experimental setup and design

The experimentation was carried out using compartmented pots including rhizosphere and root-free zones (mesh bags), allowing us to test the AM fungal effect on AO both in the presence and absence of plant roots (Fig. 1). The establishment of the root-free zone was similar as in our previous experiments [30,36]. Specifically, plants were growing outsides of a 500-mL plastic cylinders with permeable walls and bottom (2-mm openings, cat. no. P00718, Annelli, Montanaso Lombardo, Italy) covered with a polyamide mesh fabric (40-μm mesh openings; commercially available as Uhelon 130T; Silk & Progress, Brněnec, Czech Republic). The pots (20 cm × 11 cm × 11 cm, h × l × w) contained 2 L of cultivation substrate. The substrate was a mixture (45:45:10, v:v:v) of autoclaved quartz sand, autoclaved zeolite (2.5–4 mm grain size), and γ-irradiated (25 kGy) soil from Litoměřice, Czechia [37]. Physicochemical properties of the substrate have been described previously [30] and also in Supplemental Material. Aliquots of the soils from the different fields (200 g each) were added to the mesh bags (made of 40 μm nylon fabric), sealed with zip-ties and placed inside the cylinders. The edge of the cylinders was at the height of the rim of the pots, i.e., slightly higher than the substrate level. Two replicate pots were established for each AM fungus-inoculated and non-inoculated treatments with each sterilized soil (Experiment 1) and four replicate pots were included for each AM fungus-inoculated and non-inoculated treatments with each unsterile field soil (Experiment 2), resulting in 96 pots altogether (please see Supplemental Material for more details on the experimental design).

The AM fungal inoculum (i.e., living AM fungal biomass devoid of any plant roots and other microorganisms) was obtained from monoxenic liquid cultures of *Rhizophagus irregularis* isolate LPA9, which had been produced for 6 months prior to the experiment described here in association with *Cichorium intybus* Ri T-DNA-transformed roots [38]. There were approximately 30,000 AM fungal spores and uncounted hyphal fragments added to every AM-fungal inoculated pot 2 cm below the surface (approximately 15 mg dry weight of the AM fungal biomass added per pot). After soils and substrates as well as the AM fungal inoculum were applied to their designated zones, 50 seeds of *Andropogon gerardii* (Jelitto Staudensamen, Schwarmstedt, Germany) were sown 1 cm below the substrate surface. All pots were arranged randomly (i.e., completely randomized design), and the positions of the pots were rotated (i.e., swapped mirror wise) twice during the glasshouse incubation to mitigate variation of environmental conditions in the greenhouse (see Supplemental Material – Greenhouse conditions).

Small amount of deionized water was added using a water nebulizer twice a day until plant germinated. After plant emergence (approximately 3 days after sowing), deionized water was applied once a day to maintain the substrates at 65%–80% of their water holding capacity (estimated gravimetrically). At day 35, 65 mL of Long Ashton nutrient solution with reduced P concentration (containing 0.52 mg P in form of orthophosphate) and full concentration of nitrate (10.9 mg N) was added to the central cylinder of each pot. This continued every week, supplying 54.5 mg N per pot until the end of the experimentation. Throughout the duration of the two experiments (lasting 70 days), the pots were incubated in the greenhouse of the Institute of Microbiology in Prague, Czechia. More details about the greenhouse incubation parameters (i.e., temperature and light intensity) have been included in the Supplemental Material (Glasshouse conditions).

### 2.3 Harvest and measurements

At day 70, plant biomass and soil/substrate samples were collected from both Experiments 1 and 2. Plant roots were extracted from the substrate by shaking, then washed thoroughly under tap water, and rinsed briefly under deionized water. Root samples were split to aliquots for staining and drying, both weighed fresh, and the roots for drying weighed again after drying at 65 °C for 3 days. Similarly, shoots were dried, and their dry weights per pot determined. Root samples for staining were digested in 10 % KOH at 80 °C for 30 min, rinsed with water, incubated in 3 % HCl at room temperature for 30 min, and then transferred (with no further rinsing) to 0.05 % Trypan blue in lactic acid: glycerol: water (1 : 1: 1, v/v/v) and stained at 80 °C for 4 h in a water bath. Finally, the roots were incubated overnight in the lactic acid: glycerol: water mixture at room temperature. The extent of root length colonized by hyphae, arbuscules and vesicles was determined on stained root samples according to the method described previously [39], recording 100 root intersects per sample at 200 × magnification.

Representative substrate and soil samples were collected from rhizosphere and the mesh bags, respectively, (approx. 20 g fresh weight per sample) and dried at 65 °C for 3 days. Both plant and substrate samples were then pulverized using MM200 ball mill (Retsch, Haan, Germany) at 25 Hz for 2 min, employing two stainless steel balls (10 mm diameter) per sample. Total phosphorus (P) content in plant shoots and roots (using samples of 0.1 g each) were determined by the malachite green method [40] following incineration and hot HNO_3_ extraction as described previously (Arbuscular Mycorrhiza Stimulates Biological Nitrogen Fixation in Two Medicago spp. through Improved Phosphorus Acquisition). The total N in the plant biomass (2 mg) and substrates/soils (20 mg) were analyzed using the Flash EA 2000 elemental analyzer (Thermo Fisher Scientific, Bremen, Germany).

### 2.4 DNA extraction and qPCR

Root (∼10 mg dry powder) and substrate/soil (∼600/250 mg dry powder) samples were used to extract DNA using the Plant DNeasy kit (Qiagen, Venlo, Netherlands) and Dneasy PowerSoil kit (Qiagen), respectively. To determine DNA extraction efficiency, an internal DNA standard containing 2 × 10^10^ gene copies of cassava mosaic virus [41] were added to each sample prior to DNA extraction.

Specific primers (with or without a TaqMan probe) were used to measure ribosomal RNA abundances separately for the inoculant AM fungus (*R. irregularis*), bacteria, (nonmycorrhizal) fungi, and protists, and the abundance of ammonium monooxygenase (*AmoA*) gene for AOB and AOA, as detailed previously [30], using the LightCycler 480 II instrument (Roche, Rotkreuz, Switzerland). For calibration, dilution series were prepared from the relevant amplicons as described previously [30,41]. The qPCR quantification was carried out in 96-well plates using a 20-μL final reaction volume. Either Luna universal probe qPCR master mix (M3004; for assays including a probe) or Luna universal qPCR master mix (M3003; for assays without a probe were used, both supplied by the New England Biolabs, Ipswich, MA, USA. Fluorescence was recorded in the SYBR green/fluorescein color channel.

### 2.5 Statistical analyses

The response ratio (RR) was calculated to quantify the AM fungal inoculation effect in comparison to non-inoculated control treatment. For satisfying ln-ratio calculation, values of selected variables (see Table 1, Table 2) were added (transformed) with +1 before further calculations. Relevant non-inoculated treatments (controls, i.e., pots with the same soil added to the mesh bags but with plants not inoculated with *R. irregularis*) were used for calculation of the RR for each soil treatment separately, using a framework outlined previously [42]:

$$RR=\ln\frac{\text{Individual}\;\text{value}\;\text{of}\;\text{AM}\;\text{fungal}\;\text{treatment}\;}{\text{Mean}\;\text{value}\;\left(\text{relevant}\;\text{non}-\text{inoculated}\;\text{treatment}\right)}$$

**Table 1:** Relative responses to inoculation with *Rhizophagus irregularis* LPA9 with respect to plant and microbial parameters in the rhizosphere (filled with artificial substrate) and mesh bags (filled with unsterile cropland or grassland soils) in Experiment 2. One-sample Wilcoxon signed-rank test scrutinized whether medians of the different parameters significantly differed from zero. Positive and negative median values indicate promotion and suppression, respectively, in the inoculated vs. non-inoculated treatments. Rhizophagus: Abundance of *Rhizophagus irregularis* measured by targeting mitochondrial large ribosomal subunit (LSU) gene (mt5 marker); Funneliformis: *Funneliformis mosseae* measured by nuclear LSU gene abundance (MOSS marker); Claroideoglomus: *Claroideoglomus claroideum* measured by nuclear LSU gene abundance (CLAR marker); AOB CTO: 16S rRNA gene abundance of ammonia oxidizing bacteria; AOA *amoA* C: *amoA* gene abundance of ammonia oxidizing archaea (supposedly specific for *Crenarchaeota* clade). Statistically significant (p < 0.05) differences of medians from zero shown in bold.

Compartment	Parameter	Median	p-value
Plant (shoots + roots)	Plant biomass	0.460	**<0.001**
Plant (shoots + roots)	Plant phosphorus	0.677	**<0.001**
Plant (shoots + roots)	Plant nitrogen	0.127	**<0.001**
Plant roots	Hyphae^a^	0.461	**0.008**
Plant roots	Arbuscules^a^	−0.275	0.594
Plant roots	Vesicles^a^	−0.031	0.242
Plant roots	Rhizophagus^a^	0.144	0.074
Plant roots	Funneliformis^a^	−3.66	**0.002**
Plant roots	Claroideoglomus^a^	not detected	
Rhizosphere	Rhizophagus^a^	1.98	**<0.001**
Rhizosphere	Funneliformis^a^	−0.903	**0.024**
Rhizosphere	Claroideoglomus^a^	−4.68	**<0.001**
Rhizosphere	Fungal ITS rRNA gene	0.330	**0.021**
Rhizosphere	Protist 18S rRNA gene	0.124	0.379
Rhizosphere	AOB CTO	−1.727	**<0.001**
Rhizosphere	AOA *amoA* C	−0.437	**0.002**
Mesh bags	Rhizophagus^a^	1.83	**0.002**
Mesh bags	Funneliformis^a^	−0.726	**0.006**
Mesh bags	Claroideoglomus^a^	0.059	0.415
Mesh bags	Fungal ITS rRNA	−0.159	**<0.001**
Mesh bags	Protist 18S rRNA	−0.069	0.087
Mesh bags	AOB CTO	−0.231	**<0.001**
Mesh bags	AOA *amoA* C	−0.021	0.733

a All values for this parameter transformed x_1_ = x+1 before calculating mycorrhizal responses.

**Table 2:** Differences in plant/microbial responses to inoculation with *Rhizophagus irregularis* LPA9 in the rhizosphere and mesh bags in Experiment 2 as regards the land use (cropland vs. grassland) of the soils filled in the mesh bags, as per the non-parametric Kruskal-Wallis test. Positive and negative median values indicate promotion and suppression, respectively, in the inoculated vs. non-inoculated treatments. Rhizophagus: *Rhizophagus irregularis* abundance measured by mt5 marker; Funneliformis: *Funneliformis mosseae* measured by MOSS marker; Claroideoglomus: *Claroideoglomus claroideum* measured by CLAR marker (see legend to Table 1 for details); AOB CTO: 16S rRNA gene abundance of ammonia oxidizing bacteria; AOB *amoA*: *amoA* gene abundance of ammonia oxidizing bacteria; AOA *AmoA* T: *amoA* gene abundance of ammonia oxidizing archaea (supposedly specific for Thaumarchaeota clade); AOA *amoA* C: *amoA* gene abundance of ammonia oxidizing archaea (supposedly specific for Crenarchaeota clade). Statistically significant (p < 0.05) differences shown in bold.

Compartment	Parameter	Cropland	Grassland	p-value
Plant (shoots + roots)	Plant biomass	0.311	0.570	**0.002**
Plant (shoots + roots)	Plant phosphorus	0.404	1.14	**<0.001**
Plant (shoots + roots)	Plant nitrogen	0.097	0.163	0.474
Plant roots	Hyphae^a^	−0.093	2.01	**0.005**
Plant roots	Arbuscules^a^	−0.840	1.73	**0.001**
Plant roots	Vesicles^a^	−0.396	1.74	**0.005**
Plant roots	Rhizophagus^a^	−0.817	3.41	**<0.001**
Plant roots	Funneliformis^a^	−1.03	−7.10	0.234
Plant roots	Claroideoglomus^a^	Not detected		
Rhizosphere	Rhizophagus^a^	0.563	4.19	**<0.001**
Rhizosphere	Funneliformis^a^	−2.63	−0.903	0.263
Rhizosphere	Claroideoglomus^a^	−5.25	−3.24	0.062
Rhizosphere	Bacterial 16S rRNA gene	0.261	0.263	0.970
Rhizosphere	Fungal ITS rRNA gene	0.207	0.406	0.498
Rhizosphere	Protist 18S rRNA gene	0.055	0.180	0.940
Rhizosphere	AOB CTO	−1.39	−1.81	**0.042**
Rhizosphere	AOB *amoA*	−0.761	−1.34	0.065
Rhizosphere	AOA *amoA* T	−0.129	−0.532	**0.018**
Rhizosphere	AOA *amoA* C	−0.318	−0.555	0.113
Mesh bags	Rhizophagus^a^	−0.382	4.80	**<0.001**
Mesh bags	Funneliformis^a^	−0.726	−1.04	0.678
Mesh bags	Claroideoglomus^a^	0.277	0.000	0.820
Mesh bags	Bacterial 16S rRNA	0.007	−0.062	0.152
Mesh bags	Fungal ITS rRNA	−0.193	−0.150	0.498
Mesh bags	Protist 18S rRNA	−0.044	−0.076	0.706
Mesh bags	AOB CTO	−0.158	−0.409	0.113
Mesh bags	AOB *amoA*	−0.088	−0.341	0.090
Mesh bags	AOA *amoA* T	0.034	−0.048	0.274
Mesh bags	AOA *amoA* C	0.032	−0.047	0.366

a All values for this parameter transformed x_1_ = x+1 before calculating response ratios.

The RR < 0 implies suppression due to AM fungal inoculation, RR > 0 implies promotion due to AM fungal inoculation, and RR = 0 implies no effect of the AM fungal inoculation on the specific variable.

Fulfilling assumptions of ANOVA were checked for the different data (either untransformed or the RR values) by QQ-plot, Shapiro-Wilk test for normality, Levene's test for homogeneity of variances, and the possible inter-dependencies using a scatterplot or autocorrelation plots by “car” package in R 4.2.2 (R Core Team 2022). When conditions for ANOVA were fulfilled (such as in sterile soils in mesh bags), two-way analyses of variance (ANOVA) were performed to determine the effects of land use (cropland vs. grassland), mycorrhizal inoculation (with and without *Rhizophagus irregularis* inoculum), and their interactions on variables such as plant biomass, nutrient acquisition, AM fungal colonization of roots, and copy numbers of the microbial genes/intergenic spacers. When conditions of ANOVA for RR were not met, 1) one-sample Wilcoxon signed-rank test was used to answer whether the medians of RR were significantly different from a hypothesized value (mu = 0); and 2) Non-parametric Kruskal-Wallis test was applied to determine if there were any significant differences in RR with respect to land use of soil origin (i.e., cropland vs. grassland).

## 3 Results

### 3.1 Validation of successful AM fungal inoculation (Experiment 1 – sterile soils in mesh bags)

**Fig. 2:**
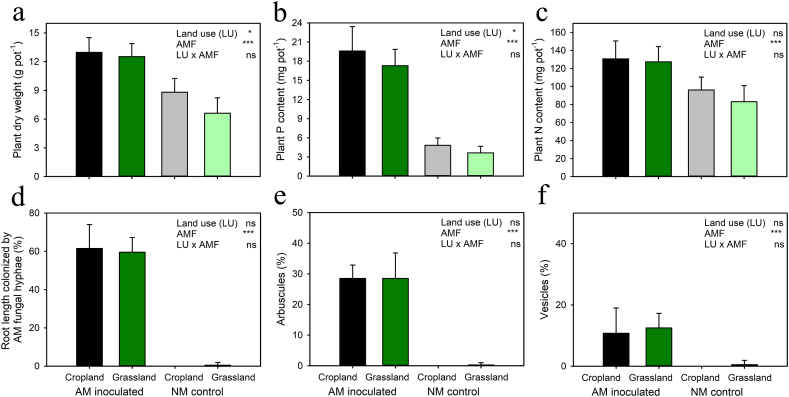
Plant biomass production (shoots and roots combined), P content and N content in plant biomass per pot, and root colonization by arbuscular mycorrhizal (AM) fungus, assessed microscopically, in response to plant inoculation with the AM fungus *Rhizophagus irregularis* LPA9 (AMF), and the land use (LU) of the γ-rays sterilized soils (cropland or grassland, n = 4) placed into the mesh bags of Experiment 1. Each bar represents a mean ± 1 SD of values from 8 independent pots, with duplicate pots included for each field soil (i.e., one soil of each land use type collected in each of the four locations). Two-way ANOVA scrutinized the significance of the experimental factors and their interaction for each particular parameter. p < 0.001 (***), p < 0.05 (*), p ≥ 0.05 (ns).

**Fig. 3:**
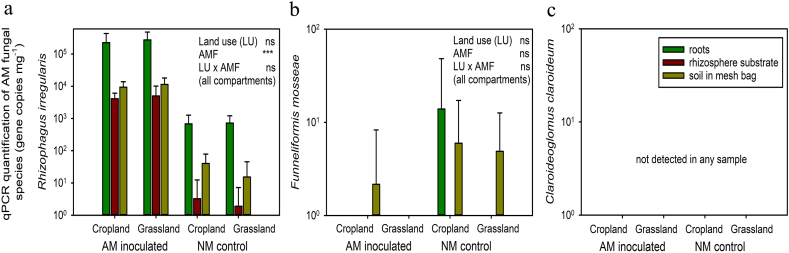
Colonization of *Andropogon* roots, potting substrate in the rhizosphere, and soils in the mesh bags of Experiment 1 by *Rhizophagus irregularis*, *Funneliformis mosseae*, and *Claroideoglomus claroideum*, as per the quantitative real-time PCR with species-specific markers. The effects of AM fungal inoculation with the AM fungus *Rhizophagus irregularis* LPA9 (AMF) and land use (LU, cropland vs. grassland) are shown of the soil used to fill the mesh bags. Please note the logarithmic scale of the y-axis. Two-way ANOVA scrutinized the significance of the experimental factors and their interaction for each particular parameter. p < 0.001 (***), p ≥ 0.05 (ns).

When the soils in mesh bags were sterilized prior to administering them to the pots, AM fungal inoculation consistently increased plant biomass and nutrient (P and N) acquisition with only a limited effect of soil legacy on the various measured parameters (Fig. 2a, b, c). Soils collected from croplands caused plants to absorb more P than the soils from grassland, and this also had a significant (stimulatory) effect on plant growth (Fig. 2a and b). In the non-inoculated control treatment, colonization of roots by AM fungal structures (assessed microscopically) was negligible (Fig. 2d, e, f). Likewise, colonization of roots, rhizosphere, and mesh bags with inoculant *Rhizophagus irregularis* as per qPCR was at least two orders of magnitude higher in inoculated as compared to the non-inoculated pots (Fig. 3a). Detection of other AM fungi which might (or their DNA) be present in the field soils such as *Funneliformis* or *Claroideoglomus* was negligible across all the samples (Fig. 3b and c, please note the logarithmic scale of y-axis).

### 3.2 a.m. fungal inoculation effects – plant growth and microbiomes (Experiment 2 – unsterile soils in mesh bags)

The AM fungal inoculation significantly increased plant biomass and nutrient (N and P) acquisition in Experiment 2 (Table 1). The percentage of root length colonized by AM fungal hyphae was also significantly promoted by AM fungal inoculation (Table 1), but this was not the case for the fractional root length colonization by arbuscules or vesicles across the different soil treatments (Table 1).

**Fig. 4:**
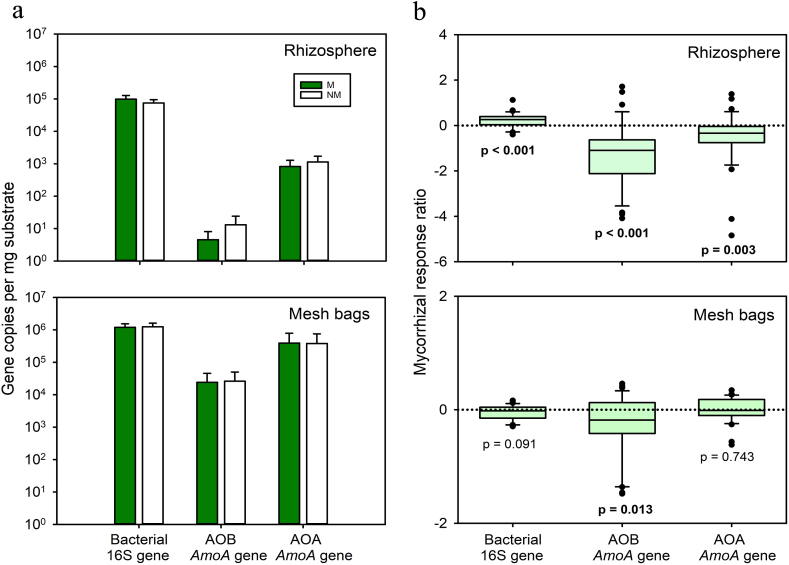
Gene copies of bacterial 16S rRNA, AOB *amoA* gene, and AOA *amoA* gene (supposedly specific for Thaumarchaeota clade) as affected by inoculation with arbuscular mycorrhizal (AM) fungus *Rhizophagus irregularis* LPA9 (M) compared to a non-inoculated (NM) control treatment in rhizosphere and in the mesh bags (left, gross average across all 8 unsterile soils in Experiment 2, n = 32, please note the logarithmic scale) of the Experiment 2. Response ratio (right, n = 32) of bacterial 16S rRNA, AOB *amoA*, and AOA *amoA* (Thaumarchaeota) gene abundances to inoculation with *Rhizophagus irregularis* in the rhizosphere (filled with an artificial substrate) and mesh bags (filled with unsterile cropland or grassland soils). The response ratios are displayed as medians, 25% and 75% percentiles (boxes) and 5% and 95% percentiles (error bars) and were calculated separately for each particular soil, using individual M values and average of the relevant NM value. The p-values refer to nonparametric one-sample Wilcoxon signed-rank test for differences of sample median from zero. Statistically significant (p < 0.05) shifts in abundance of the microbes due to AM fungal inoculation are shown in bold.

In the rhizosphere (filled with an artificial substrate), both AOB and AOA were suppressed by AM fungal inoculation (Table 1, Fig. 4b). In the mesh bags filled with unsterile field soils), only AOB were significantly suppressed by AM fungal inoculation (Table 1, Fig. 4b), whereas AOA were not affected by the AM fungal inoculation (Table 1, Fig. 4b).

At the same time, the AM fungal inoculation significantly promoted abundances of bacterial 16S rRNA gene and fungal internal transcribed spacer (ITS) of the rRNA operon in the rhizosphere, whereas the abundance of protistan 18S rRNA gene was not affected by the inoculation. In the mesh bags, bacterial and protistan abundances were not affected by the AM fungal inoculation, whereas the abundance of (non-mycorrhizal) fungi was suppressed by the AM fungal inoculation (Table 1, Fig. 4a).

The inoculation with *Rhizophagus irregularis* increased its abundance in the rhizosphere and in the mesh bags across all the soils (though not in the roots) as compared with the non-inoculated control treatment (Table 1). Abundance of *Funneliformis mosseae* was suppressed both in plant roots, rhizosphere and in the mesh bags due to inoculation with the *R. irregularis* (Table 1). *Claroideoglomus claroideum* was not detected in the roots, its abundance was suppressed in the rhizosphere, and not affected in the mesh bags due to inoculation with *R. irregularis* (Table 1).

### 3.3 Soil legacy effects in Experiment 2 – croplands vs. farmlands

**Fig. 5:**
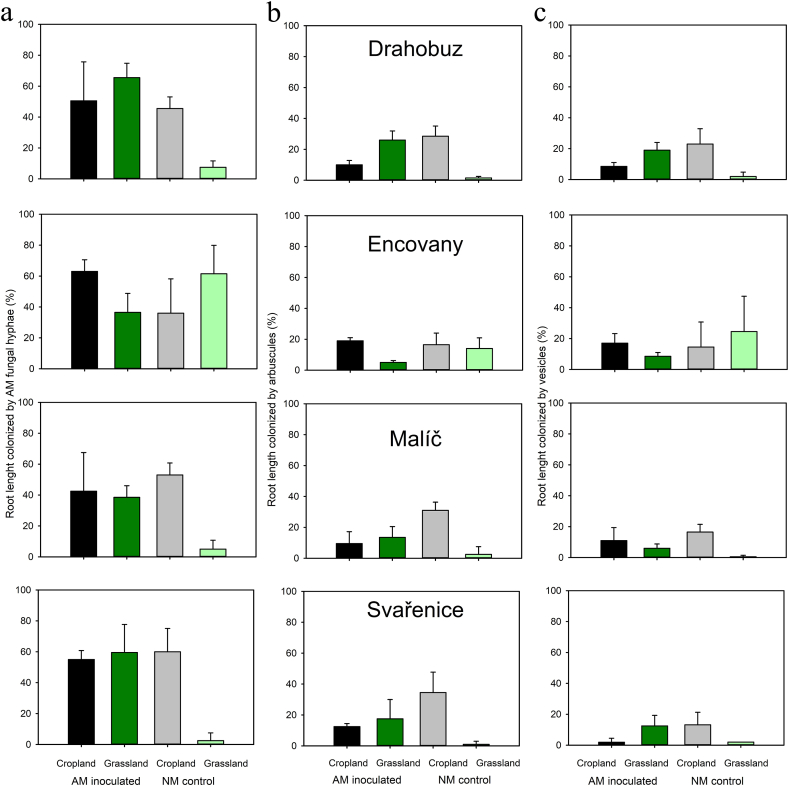
Percentages of plant root length colonized by hyphae, arbuscules, and vesicles of the arbuscular mycorrhizal (AM) fungi, assessed microscopically in Experiment 2, as affected by *Rhizophagus irregularis* LPA9 inoculation (AM inoculated) vs. non-inoculated (NM control) treatment. Soils to fill the mesh bags were collected from both croplands and grasslands of the four locations (Drahohuz, Encovany, Malíč, and Svařenice) in Czechia. Bars represent means and standard deviations (n = 4).

In the absence of AM fungal inoculation, the percentage of root length occupied by AM fungal hyphae, arbuscules, and vesicles was generally higher when mesh bags were added with unsterile cropland soils compared with grassland soils (with exception of one site, the Encovany, where the values were comparable, Fig. 5b). On the other hand, upon AM fungal inoculation of the plants, the percentage of root length colonized by AM fungal hyphae, arbuscules, and vesicles was comparable for grassland and cropland soils added to the mesh bags (Fig. 5a, b, c). This resulted in large contrasts in root colonization parameters in pots amended with mesh bags containing unsterile soils with respect to soil legacy due to AM fungal inoculation – the effects were invariably positive for grasslands whereas they were close to zero or negative for croplands (Table 2).

The extent of mycorrhizal benefits thus depended on the legacy of soils added to the mesh bags. There was greater effect of mycorrhizal inoculation on plant biomass for grassland as compared to cropland soils (Table 2) and the contrast was even stronger for plant P content (Table 2). On the other hand, no difference between previous land use of soils used to fill the mesh bags was detected for mycorrhizal benefits with respect to plant N uptake (Table 2).

**Fig. 6:**
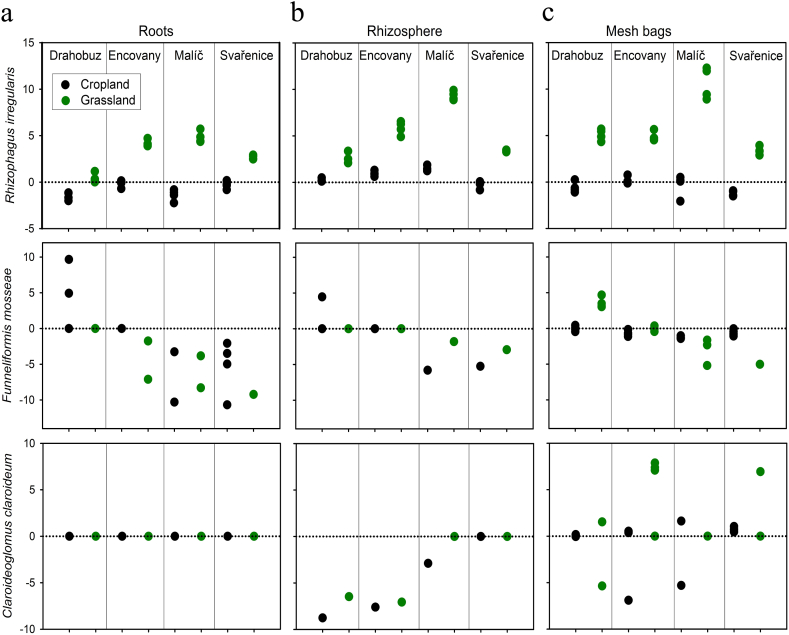
Response ratios of the abundance of three arbuscular mycorrhizal (AM) fungal species (*Rhizophagus irregularis*, *Funneliformis mosseae*, and *Claroideoglomus claroideum*) in plant roots, rhizosphere substrate and mesh bag soils of Experiment 2, as per the quantitative real-time PCR, to inoculation with *R. irregularis* LPA9 as compared to the non-inoculated treatment, both supplied with mesh bags containing unsterile field soils. Individual values are shown, which were calculated separately for the roots, rhizosphere, and mesh bags using individual measurements from AM fungus-inoculated pots and mean values of the relevant non-inoculated control treatment. When two or more (replicate) values for one soil treatment were identical, they are shown as a single datapoint.

The AM fungal inoculation increased the abundance of *Rhizophagus irregularis* in all pot compartments when grassland soil was in the mesh bags as compared with cropland soils (Table 2, Fig. 6a, b, c). On the other hand, the abundance of indigenous AM fungal genera which were not supplied with the AM fungal inoculum (e.g., *Funneliformis* and *Claroideoglomus*) in any of the pot compartments was not significantly affected by the soil legacy (Table 2), although their abundance was generally suppressed by AM fungal inoculation, particularly in the rhizosphere (Table 1, Fig. 6b).

Besides, the AM fungal inoculation had a stronger suppression effect when mesh bags were filled with grassland soil (as compared to cropland soils) on 16S gene abundance of AOB and on the abundance of AOA as quantified by Thaumarchaeota-specific *AmoA* gene motif in the rhizosphere, with no detectable effect of soil legacy on the different microbial guilds in the mesh bags (Table 2).

## 4 Discussion

In an experimental work like the one presented here it is important to ensure successful AM fungal inoculation, root colonization and hyphal proliferation in the root-free compartments. For this reason, and also to verify that the physico-chemical properties of the different soils did not hamper development of our *R. irregularis* isolate (although nutrient availability usually was elevated following soil sterilization, see Supplemental Material for soil analyses), it was important to establish pots with previously sterilized soils added to the mesh bags, i.e., the Experiment 1. Besides, the growth and nutrition of experimental plants indicated that not only the AM fungal hyphae were efficient in foraging for nutrients in the mesh bags, but also that the cropland soils supplied significantly larger P (though not N) amounts to the plants as compared to the grassland soils. This subsequently translated into greater biomass production of AM fungus-inoculated plants with cropland soils added to the mesh bags as compared to pots added with grassland soils. These results are consistent with the previously demonstrated high residual P reserves in cropland soils [43].

In our study, we made an important discovery that sheds light on a specific role of AM fungi in interacting with soil microbes in general and the AO in particular, in agricultural soils. Specifically, we found that AM fungi suppressed indigenous AOB communities in the mesh bags (which were inaccessible to plant roots), and that this suppression was independent of soil identity (although the different soils differed markedly in the size of AOB and AOA communities from each other, see Supplementary Material for details) or land use legacy. This finding confirms our first hypothesis and highlights the need to distinguish between direct AM fungi-induced effects and more general AM-symbiosis mediated effects on N-cycling processes, of which the latter could also include modifications of root systems and/or exudation [44,45]. Previous studies did not exclude root interference from the interactions between AM fungi and the AO [27,29], although these may well have a different outcome as also illustrated in Fig. 4b here. In contrast, our mesh bags allowed to exclude root interference, and this appeared important as the roots may have inhibited AO by exuding biological nitrification inhibitors [46,47] or by competing with AO for ammonia or possibly for other (mineral nutrient) resources. Our findings thus provide novel insights into the complex interactions between AM fungi and AO and highlight the importance of careful experimental design in studying these interactions. Why the AM fungus did not suppress AOA in the selection of soils included in our experimentation remains unclear – likewise it remains unclear whether the AOA play any important role in nitrification in agricultural (aerated and mostly neutral to alkaline) soils in a temperate zone [48,49].

Our study thus revealed an emerging trend that to some extent contradicts previous research. While no effect of AM fungi was found on the potential nitrification rates or AOB in previous study [29], our study showed that AM suppressed AOB in a selection of agricultural (top)soils within mesh bags where plant roots were excluded. Similar results as obtained in this current study were already reported previously, but only for artificial substrates filling root-free patches [30,36]. Additionally, we observed that the suppression exerted by the AM fungi on AOB was not likely dependent on soil fertility, as our soils ranged from low to high fertility (N content ranging from 0.11 % to 0.35 %, and C content ranging widely from 1.6 % to 9.7 %). This finding challenges the hypothesis that soil fertility plays a crucial role in the effects of AM fungi on AOB [27].

Interestingly, we also found that the AOA may not be as responsive to AM fungi as the AOB, which was the case in our mesh bags but not in the rhizosphere. Significant suppression, although a weaker one as compared to the AOB, was observed for AOA communities due to AM fungal inoculation in the rhizosphere. This could be due to indirect mycorrhizal effects mediated by roots (which might change morphologically but also metabolically as compared to non-mycorrhizal roots [45]. Alternatively, this could be due to use of artificial cultivation substrate (containing just 10% of soil by volume, and the rest composed of sand and granular zeolite) to fill the rhizosphere compartment in our pots. In line with the latter notion, our current observation contradicts a previous finding that AOA abundance was not affected by AM fungi in mesh bags containing artificial substrates and ^15^N-labelled litter [30].

AOA abundance was found strongly controlled by N content of substrate, such as urea addition into the rhizosphere [50]. Previously, plants were found to have a key role in controlling the fate of ammonium-N in N-deficient ecosystems [51]. The rhizosphere in our study was also N- (and particularly ammonia-) deficient compared with the mesh bags containing agricultural soils. The nutrient solution applied regularly into our experimental pots contained exclusively nitrate-N (see Supplementary Material - Long Ashton nutrient solution). The rhizosphere (i.e., the root) effect may be even stronger than the AM fungal effect because plant roots could suppress AO abundance by competing for ammonia [27]. The suppression of AOA by AM fungi in the rhizosphere may also be due to interplay with other soil microorganisms, as suggested recently [52] or as demonstrated in another study [38].

In contrast to our expectations (hypothesis 2), we could not uncover any consistent effect of AM hyphal ingrowth into the mesh bags on resident AO communities in the different soils as depends on the soil legacies (cropland vs. grassland). We did, however, demonstrate that indigenous AM fungi from cropland soils were generally more capable of germinating/proliferating from root and/or hyphal fragments, and subsequently colonizing roots of our experimental *Andropogon* plants across two layers of finely meshed nylon fabrics. Such capacity was much lower to nil for indigenous AM fungi from grassland soils – with a notable exception of AM fungal communities from a grassland in Encovany site (Fig. 5a). Our preliminary molecular genetic analyses of indigenous AM fungal communities in the soils used in the pot experiments described above (data not shown here) indicated much higher relative abundance of unidentified *Glomus* p. in that particular grassland soil, contrasting with the three other grassland soils. In spite of the fact that many *R. irregularis*-uninoculated pots added with unsterile cropland soils into the mesh bags showed AM fungal colonization in the roots, rhizosphere and the mesh bags (please see Supplemental Material for details), the general trend of suppression of the AOB communities in the mesh bags still held on. This possibly indicates that the AM fungal colonization in the non-inoculated pots developed later or that the AM fungal networks in the mesh bags were less proliferous than in case of AM fungus-inoculated pots, or that the inoculant AM fungus was particularly effective at suppressing the AO communities as compared to the indigenous AM fungi.

## 5 Conclusions

This study primarily demonstrated that ingrowth of AM fungal hyphae could suppress the abundance of indigenous AOB but not AOA in a range of agricultural soils, not depending on the legacy (cropland vs. grassland) of such soils. Furthermore, our research highlighted that indigenous AM fungi, particularly those originating from croplands, were more effective in escaping from the mesh bags and colonizing plant roots than those originating from grasslands. The cropland AM fungi (though obviously not the *Claroideoglomus*, see Fig. 6 and Supplementary Material for details) regularly made it through two layers of a fine (40 μm) nylon fabric separating the soil added to the mesh bags from the plant roots. Besides addressing this technical issue (possibly by temporal shift of plant inoculation and mesh bag placement), future research should focus on understanding the underlying mechanisms behind the differential effects of AM fungus/fungi on the different members of AOB and AOA communities, as well as the conditions under which AOA may be affected by AM fungi (such as observed here in the rhizosphere and also elsewhere [53].

## CRediT authorship contribution statement

**Daquan Sun:** Writing – review & editing, Writing – original draft, Methodology, Investigation, Formal analysis. **Martin Rozmoš:** Validation, Methodology, Investigation. **Michala Kotianová:** Validation, Methodology, Investigation, Data curation. **Hana Hršelová:** Validation, Methodology, Investigation, Data curation. **Jan Jansa:** Writing – review & editing, Validation, Supervision, Funding acquisition, Conceptualization.

## Declaration of competing interest

The authors declare that they have no known competing financial interests or personal relationships that could have appeared to influence the work reported in this paper.
